# Heterogeneity in English as a Foreign Language: Skills Among Norwegian 6th Graders with Dyslexia—The Impact of Language Comprehension and Processing Profiles

**DOI:** 10.3390/brainsci15111230

**Published:** 2025-11-15

**Authors:** Turid Magnhild Helland, Randi Kaasa, Wenche Andersen Helland

**Affiliations:** 1 Department of Biological and Medical Psychology, University of Bergen, 5020 Bergen, Norway; wenche.helland@uib.no; 2 Logopaedic Clinic, Haukeland University Hospital, 5009 Bergen, Norway; kaasa.randi@gmail.com; 3 Department of Research and Innovation, Fonna Health Trust, 5504 Haugesund, Norway

**Keywords:** dyslexia, EFL, subgroups, comprehension, pragmatics, reading, spelling, visual timing, auditory timing

## Abstract

**Background:** English as a first foreign language (EFL) is often difficult for students with dyslexia. This study maps a broad range of EFL verbal and literacy skills in 6th graders with dyslexia compared to a control group. **Methods:** Participants were 127 controls (CON) and 60 students with dyslexia (DYS), split into dys+ and dys− by their comprehension scores. They were tested with “The English 2 Dyslexia Test” containing seven subtests within three domains: Sentences, Pragmatics, and Literacy. The data were analysed in *Part 1*: domains and groups, and in *Part 2*: linguistic skills and spelling by groups. **Results:** *Part 1*. CON scored better than DYS on all tests. However, the differences between the two subgroups, dys+ and dys−, were larger than expected. Dys+ scored in line with CON on several tests, while dys− scored below CON on all tests and lower than dys+ on all except for spelling. *Part 2*. Minor differences were seen between CON and dys+ in linguistic skills, and both CON and dys+ scored higher than dys−. Spelling was scored by the number of graphemes. CON scored higher than both dys+ and dys−, with no difference between the subgroups. **Conclusions:** The results were discussed in accordance with neurocognitive theories of the auditory and visual timing systems. The overall low scores in dys− were mainly attributed to auditory processing problems, while the specific low spelling scores in dys+ were mainly attributed to visual processing problems. More research is needed on how the behavioural patterns in the two dyslexia subgroups relate to neural correlates in the meeting between EFL and different L1 language typologies and orthographies.

## 1. Introduction

English as a first foreign language (EFL) is common in schools across many countries and is essential for education, working life, and interaction with people around the world. Within primary education, 83.9% of pupils in the EU were learning EFL in 2022 [1]. International pedagogical views focus on communicative language competence with less focus on grammatical teaching [2,3].

Foreign language teachers face a great variety of students in the classroom [4]. It is common knowledge that EFL is particularly difficult for people with dyslexia [5,6,7,8,9,10,11]. According to the classic causal model by Morton & Frith [12], dyslexia can be understood at a symptomatic, a biological, and a cognitive level, which are all influenced by the environmental level. The definition of the British Dyslexia Association [13] contains the four levels of the causal model (authors’ comments in parentheses):

*Dyslexia is a specific learning disability that primarily inhibits reading and writing development and other language-related skills (symptomatic level). The difficulty is usually congenital and lasts a lifetime (biological level). Typical characteristics are difficulties with phonological processing, rapid naming, working memory, processing speed, and automation of skills that deviate from the person’s other cognitive skills (cognitive level). Conventional teaching methods are usually not effective, but the consequences of these difficulties can be remedied or compensated for through adapted and specific training, including the use of information technology and supportive counselling* (environmental level).

**Symptoms** of dyslexia change with age and literacy development. According to Frith [14] and Ehri [15], there are three stages in typical reading and writing development: the pre-literacy stage, ahead of formal literacy training; the emergent literacy stage, when the child receives formal literacy training; and, finally, the literacy stage, when automatized, functional literacy is achieved. In most European countries, EFL is introduced in the first years of primary school, i.e., in the emergent literacy stage, at the same time as the reading and writing code is to be cracked. Studies show that literacy achievement is slower in deep orthographies compared to shallow orthographies [16].

At the **biological** level, there is a consensus that dyslexia is associated with aberrant structures, especially in the left hemisphere language network, but with variations according to language typologies and individual compensatory mechanisms and age [17,18,19,20,21,22,23]. Comparison between monolinguals and bilinguals shows that bilingual learning environments create patterns in the neural activity of the brain that are qualitatively different from the patterns one finds in monolingual development [24]. Furthermore, brain imaging suggests that early bilingualism activates the same areas of the brain in both languages, and that this activation is more bilateral than in monolinguals or in late bilinguals [25].

The **cognitive level** is characterized as the bridge between the symptomatic and the biological levels and is essential to clinical diagnosis. The significance of the cognitive dyslexia benchmarks listed in the BDA definition is inconclusive. They are often analysed within the Model of Working Memory, with its four main components: the phonological loop, the visuo-spatial sketchpad, the central executive, and the episodic buffer [26]. These components are fundamental to the understanding of language skills, both within the neurolinguistic and the neurocognitive domains [26,27,28,29,30,31]. For students with dyslexia, five cognitive factors play an important role in EFL assessments: phonological processing, auditory system, visual system, processing speed, and semantic lexicon [32]. In addition, motivation plays an important role in L2 learning [33,34,35]).

At the **environmental** level, individual assessment and intervention strategies are essential in minimizing the effects of dyslexia on education and social activities [36]. Important factors regarding learning a new language are age, degree of professionalism, and exposure to the language, which in turn affect brain activity through complex interactions [37,38].

In neuropsychological terms, language learning can be understood in accordance with two different principles of information processing. Bottom-up processing refers to how a flow of information is analysed from sensory integration to a higher, cognitive level. In top-down processing, complex information is processed by the pre-existing knowledge in long-term memory [39]. Speech perception abilities in infants predict their language development, and linguistic, social, and cognitive skills contribute to differences seen in their language development [40]. Language experience causes neural changes, and as neural networks develop, they make it easier for new speech elements and patterns to be learned if they are consistent with the existing patterns. However, established neural networks will interfere with and, to some degree, inhibit the learning of new languages [41]. Brain studies show bilateral differences in second language learning [42]; this is reflected in behavioural studies showing that children with L1 problems also have problems with L2 learning [43,44,45]. Speech perception and comprehension are fundamental to language learning but are less demanding than production. What is difficult, and what is easy for the individual student, is often unclear [46]. For dyslexia, our former Norwegian study showed that pupils with EFL comprehension problems had lower scores on both verbal and literacy tests compared to dyslexic pupils with no comprehension problems [47].

To analyse divergent language development in any language, it is necessary to assess how these aspects relate to different aspects of language. Linguists divide language into *form*, *content*, and *use* [48], which mutually influence each other. *Form* consists of phonology, morphology, and syntax. A phoneme is the smallest unit of speech that makes one word different from another word, i.e., the vowels in *bag* and *beg*. Phonemes are spoken sounds, while graphemes are written symbols that represent those sounds. The conjugational systems of morphology in English and Norwegian have much in common, but there are also differences, such as the third person -s in the singular present tense, regular plural inflexion in nouns with the addition of an -s, and irregular inflexion of nouns and verbs in English. Furthermore, there are differences when it comes to syntax with the use of the continuous verb form, “to do”, in interrogative and negative sentences, and little use of inversion (swapped placement of subject and verb) in English. Semantics deals with *content* and vocabulary, while pragmatics deals with language *use* in different situations. Vocabulary learning and sentence production are generally demanding in L2 learning, especially in terms of auditory functions [49,50].

The role of auditory versus visual impairment in reading and spelling has been a controversy within dyslexia research since the so-called “pioneers” observed and described the condition of “word blindness” [51,52]. Later, the main understanding of dyslexia has been on phonological awareness [53,54,55,56]. Today, dyslexia is seen as a multidisciplinary impairment where language typologies may play an important role [19,21,57,58,59]. According to Frith [60], written language requires a simultaneous perception of the word’s sound, appearance, and meaning. Hence, the relationship between phonemes and graphemes plays an important role in various orthographies [61].

**Figure 1:**
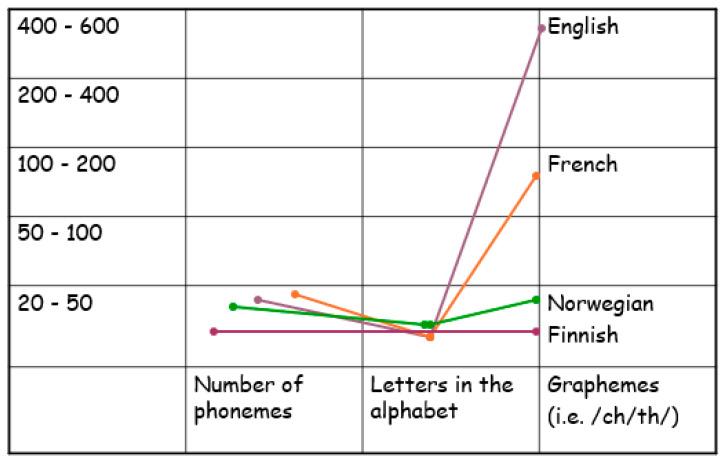
Relationship between phonemes, letters, and graphemes in Finnish, Norwegian, French, and English. From Helland (2019) [65].

English, with its deep orthography, differs greatly from shallow and semi-shallow orthographies, such as, for instance, German, Finnish, and Norwegian. In this respect, learning EFL literacy based on L1 literacy in a shallow orthography challenges visual memory of complex phoneme/grapheme correspondences in the English orthography. The Norwegian alphabet has 29 letters and 40–44 phonemes in the language, depending on the dialect, expressed by 36 graphemes [62]. The English orthography has 26 letters and 41–44 phonemes, which are expressed via 561 graphemes [63]. For instance, English words with identical pronunciation are spelled differently depending on context. Examples are “to/two/too” and “jeans/genes”. Incorrect spelling can be understood either as phonologically based, as visually based, or as a combination of the two components. As illustrated in Figure 1, English, with its deep orthography, differs greatly from shallow and semi-shallow orthographies, such as Finnish and Norwegian. Examples are words for English “cake” [keık]: “kakku” [ˈkɑkːu] (Finnish) and “kake” [ˈkɑːkə] (Norwegian), transcribed by the International Phonetic Alphabet (IPA) in brackets. Thus, learning to spell in EFL cannot solely be based on typical sound-to-letter teaching methods, but also on visual working memory [64].

According to Dehaene [66], learning a new skill involves four steps: insight into how we learn, attention, active engagement, and error feedback. As a rule, and if possible, dyslexia should be diagnosed in the individual’s first language (L1). The phonological theory of dyslexia has been criticized for being inadequate, leading to insufficient intervention methods in some students diagnosed with dyslexia [57]. For second language (L2) learning, problems should be analysed and understood based on a professional L1 dyslexia assessment. Students with dyslexia often find that the support they receive in L1 cannot be transferred to EFL, and hence they feel insecurity or anxiety when working with this subject [67]. The outcome depends not only on teaching methods in school, but also on implicit learning via mass media and popular culture outside of school [68,69,70]. A systematic literature review on gaming and EFL among students aged 11–18 found increased motivation and learning [71]. Studies also show that there is a positive correlation between gaming and learning new words. Time spent correlates with increased vocabulary, and boys score higher than girls [72,73,74]. Gaming is motivation-driven, and language learning is implicit. Although there is little research on the effect of gaming on EFL for students with dyslexia, there is reason to assume that gaming also affects their EFL skills [73,74,75,76]. This also goes for grammar learning [77]

### This Study

In this study, we wanted to investigate EFL in a group of 6th-graders with dyslexia compared to a typical group. School entrance in Norway is at age 6 within the calendar year. According to the Norwegian English Curriculum, EFL is taught from 1st grade on with emphasis on content, use, and the increased access to English on social platforms and in the media [78]. In accordance with this pragmatic view on EFL learning, our aim was to assess both verbal and literacy skills. With reference to brain studies in dyslexia showing impaired left hemisphere functions, we expected to find group differences basically related to neurolinguistic functions. In accordance with earlier referred studies on language learning, minor group differences were expected to be seen within the dyslexia group for language comprehension, while major differences were expected to be seen for language processing, which is demanding on cognitive functions typically impaired in dyslexia. This applies to linguistic form, content, and use, as well as to literacy skills, which require a simultaneous perception of the word’s sound, appearance, and meaning.

## 2. Materials and Methods

The project was approved by SIKT (Norwegian Agency for Shared Services in Education and Research; https://sikt.no/en/home, assessed on 13 June 2023) and by the school managements in the participating municipalities. A declaration of consent was obtained from the parents of all participants and from the participants themselves. Access to individual diagnostic reports in L1 Norwegian was not within the approval options of the study. The Norwegian school system is public and unitary, with a focus on student inclusion and individually adjusted teaching. EFL is compulsory from 1st grade on with a fixed curriculum and number of lessons per year for all schools (The Norwegian Directorate for Education and Training [79]).

**Participants**. A total of 187 6th-graders, 127 controls (CON) and 60 with dyslexia (DYS) from urban and rural school districts in Norway, took part in the study. According to the class, teachers, and the school administrations, none of the control subjects had received special needs education. Dyslexia was diagnosed through the school’s support systems by skilled speech and language therapists, in accordance with the standard assessment of dyslexia in Norway. Inclusion criteria for all participants were Norwegian L1; normal vision and hearing; and no known neurodevelopmental disorders (except dyslexia), intellectual disabilities, or syndromes. All subjects were within the normal range of IQ and participated in regular classes with some support due to dyslexia.

**Procedures**. The testing took place in different school districts. The subjects were assessed individually in quiet testing rooms at the subjects’ home schools by master students in logopedics, who had been trained by and were under the supervision of the project leaders. The testers were not informed in advance about who had dyslexia. Co-scoring was carried out by the testers, and the test leaders were consulted in cases of doubt.

**The English 2 Dyslexia Test** [80] is a digitalized test used on all participants in the present study. It has a handbook with instructions on test settings, detailed for each subtest and for scoring. The test battery is divided into three domains. Domain 1 concerns verbal sentence comprehension and production, Domain 2 refers to pragmatics, and Domain 3 refers to literacy. Calculated testing time is approximately 15 min for each domain, i.e., 45 min in total. Individual testing with a qualified tester is required. The test is not diagnostic but aims to assess how 6th-graders with dyslexia perform in EFL. Feedback from teachers, clinicians, and subjects is that the test does not create anxiety but rather motivation, that it has face value, and that it assesses core items for the age group in a pedagogical way. An identical, digitally updated second version of the test is available [81].

### 2.1. Domain 1 Sentences

**T1: Sentence comprehension**. The subject is presented with fifteen short sentences of high-frequency words connected to a picture series shown on the screen. The program reads aloud one sentence at a time, and the subject is to click on the picture that fits the sentence. Five syntax structures are presented: three narrative, three interrogative, three negative, three with no inversion (but with inversion in Norwegian), and three with the passive voice. The correct keystroke awards 1 point, and the wrong keystroke awards 0 points, with a maximum score of 15 points.

**T2: Model sentences**. This subtest is made up of fifteen sentences consisting of high-frequency words and with the same syntax structure as in T1: three narrative, three interrogative, three negative, three with no inversion (but with inversion in Norwegian), and three with the passive voice. The assignment begins with three example tasks. The student is presented with a picture and hears a sentence that fits the picture. Then, a new analogue picture is shown, and the student is to produce a sentence analogous to the one in the previous picture. A correct sentence awards 1 point, and an error in the sentence awards 0 points. The maximum score is 15 points.

*Additional scoring.* Each of the fifteen sentences is evaluated separately in three parts for correct morphology, correct syntax, and correct semantics. Importantly, 1 point is awarded for correct morphology, and 0 points are awarded for incorrect morphology (max score = 15 p); 1 point is awarded for correct syntax, and 0 points are awarded for incorrect syntax (max score = 15 p); and 1 point is awarded for correct semantics, and 0 points are awarded for incorrect semantics (max score = 15 p).

### 2.2. Domain 2: Pragmatics

**T3: Daily Conversation**. The program asks the subject their name, age, activities and hobbies—eight questions in all. After the testing session, the tester transcribes the responses by listening to the audio recordings and calculates words per minute, where the responses are communicative but not necessarily linguistically correct.

**T4: Picture story**. The program presents a story of four pictures to be studied for half a minute. Then, the story is to be retold verbally in English by the student. After the testing session, the tester transcribes the narration by listening to the audio recording. The test is scored according to the calculated number of words per minute, where the narrative is communicative but not necessarily linguistically correct. The maximum score achieved for T3 and T4 was 173 words per minute, which was defined as the maximum score (the 90th percentile score was 135).

**Domain 3: Literacy**. A total of 22 high-frequency function and content words are used as the basis in the three literacy tests. In each of the assignments, the words are presented in context. The words are *boy*, *girl*, *school*, *child*, *cat*, *name*, *very*, *should*, *nose*, *mouth*, *much*, *when*, *could*, *just*, *beautiful*, *many*, *then*, *what*, *house*, *little*, *than*, and *high.*

**T5: Word dictation**. The program reads a sentence, and the pupil is then asked to write the appointed word from the text. The correct orthography awards 1 point per word, and an error in the orthography awards 0 points. The maximum score is 22 points.

*Additional scoring.* Each word is scored by correct phoneme/grapheme correspondence, i.e., “school” has 6 letters but only 4 phonemes corresponding to 4 graphemes: “s-ch-oo-l”. Spelling “skul” would award 2 points out of 4 (s and l if correctly spelled). The maximum score for all graphemes is 75 points.

**T6: Reading aloud**. The pupil reads out loud ten sentences, including the selected 22 high-frequency words. After the testing session, the test leader listens to the audio recording to score the pronunciation of each of the 22 words. Communicative pronunciation awards 1 point; non-communicative pronunciation awards 0 points. The maximum score is 22 points.

**T7: Translation**. The pupil is to translate into Norwegian the same ten sentences as in T6, reading aloud. Again, the target words are the selected 22 high-frequency words. The tester listens to the audio recording, and each correct or communicative word translation awards 1 point, and each incorrect or non-communicative word translation awards 0 points. The maximum score is 22 points.

### 2.3. Groups and Subgroups

In accordance with the earlier referred studies on the significance of comprehension in language functions, DYS was split exploratory by the median score (11p) on T1, sentence comprehension, in dys+ (*n* = 29), with scores above the median score, and dys− (*n* = 31), with scores at or below the median score. Due to the testing time and participation in a larger study, Domain 2, pragmatics, was excluded from testing in one cohort (*n* = 40). Regarding gender, Chi^2^ indicated no significant differences between the number of girls versus boys, and the LSD follow-up test showed no significant differences in points (sum of all scores) by gender (Table 1). Hence, gender was not considered in the present study.

### 2.4. Statistical Analyses

Cronbach’s alpha and split-half reliability were used to assess reliability. Correlations (Product-Moment, two variable list and one variable list, pairwise) were used to assess the relationship between the seven tests.

For an overview, two-way ANOVAs within each of the three domains with a Group (CON and DYS) and repeated-measures design were reported. For group analyses, *t*-test for independent samples and one and two-way ANOVAs were used. The alpha level was set to 0.05. Significant effects of the ANOVAs were followed up by an unequal N Tukey HSD test. For effect size analyses, a calculation *t*-test for Cohen’s d was used.

Max scores were defined by ceiling scores in Domains 1 and 3, while the top attained scores (173 words/minute) were used as the max score in Domain 3.

For illustration and to sum up the results, the raw scores were transformed into percentage scores with the ceiling scores (% correct) for T1, T2, T5, T6, and T7. For T3 and T4, the highest score (173 words/minute) from the two tests was used as a 100% correct score.

The results were divided into two parts. Part 1 presents the results of the three domains, while Part 2 focuses on the two Additional scorings of T2, separated into morphology, syntax, and semantics and T5, word dictation, where the correct number of graphemes per word was scored.

## 3. Results

Part 1. Two lists of pairwise correlations (T1 comprehension by T2, T3, R4, T4, T5, T6, and T7) were all significantly positive (*p* < 0.0001). Similarly, one list of pairwise correlations between all seven tests was significant (*p* < 0.001). The data showed a Cronbach alpha reliability of 0.822 using case-wise analyses.

⁡

**A.** Overview of groups (CON and DYS) by domains. Two-way ANOVAs with group (Con vs. Dys) and repeated measures (D1: T1 vs. T2; D2: T3 vs. T4; D3: T5 vs. T6 vs. T7) separately for each domain:

### 3.1. Domain 1 Sentences

T1 comprehension and T2 model sentences. CON vs. DYS. There was an effect of Group F(1,185) = 44.802, *p* < 0.0001. and of repeated measures F(1,185) = 907.613, *p* < 0.0001. A follow-up test showed that the effect of group was due to CON (9.89) > DYS (7.12), and that of repeated measures was due to T1 comprehension (12.18) > T2 model sentences (5.81).

### 3.2. Domain 2 Pragmatics

T3 daily conversation and T4 picture story. CON vs. DYS. There was an effect of group F(1,145) = 19.717, *p* < 0.0001 and repeated measures F(1,145) = 54.569, *p* < 0.0001. A follow-up test showed that the effect of group was due to CON (82.37) > DYS (63.44), and that of repeated measures was due to T3 (63.44) < T4 (86.78).

### 3.3. Domain 3 Literacy

T5 word dictation, T6 reading aloud, and T7 translation. CON vs. DYS. There was an effect of group F(1,185) = 100.585, *p* < 0.0001, and of repeated measures: F(2,370) = 415.356, *p* < 0.0001. A follow-up test showed that the effect of group was due to CON (17.96) > DYS (13.21), and that of repeated measures was due to T6 (19.58) > T7 (17.71) > T5 (12.02).

⁡

**B.** Overview of the Seven Tests by Groups (CON vs. DYS; CON vs. dys+ vs. dys−). As can be seen in Table 2, CON scored significantly higher than DYS on all seven tests. The DYS sub-grouping differentiated this picture. First, there were no significant differences between the scores of CON and dys+ on three (T1, T3, and T4) of the seven analyses, while dys− scored significantly lower than CON on all analyses. Also, dys− scored significantly lower than dys+ on all analyses, except for T5 word dictation, with no significant difference between the two subgroups.

Table 3 shows that the effect sizes align with the t-tests and one-way ANOVAs. In D1 and D3, the differences between CON and DYS are large, and in D2, they are medium to large. In CON vs. dys+, the effect sizes are low to medium in D1 and D2, and in D3, they are large to medium. In CON vs. dys−, all effect sizes are large, while in dys+ vs. dys−, four effect sizes are large (T1, T2, T6, and T7), while two (T3 and T5) are small to medium.

**Figure 2:**
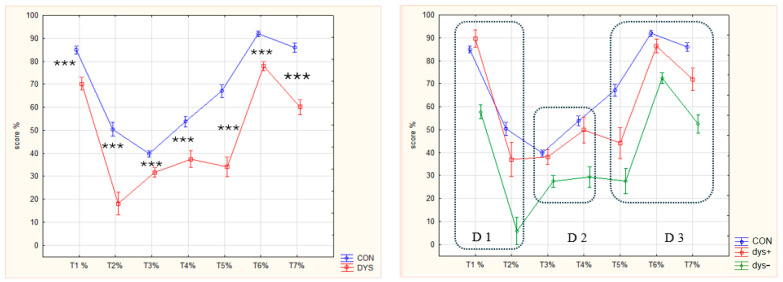
(**Left panel**) Percentage correct scores in CON and DYS with marked significant differences per test. ***: *p* < 0.001. (Right panel) CON, dys+, and dys− per domain.

Summary, Part 1. For an overview, the raw scores of each of the seven tests were transformed to per cent scores calculated from maximum scores in T1, T2, T5, T6, and T7. As for T3 and T4, the highest attained score (173 words per minute) was used as the maximum score. Figure 2 (left panel) shows the scores of CON and DYS in percentages. Figure 2 (right panel) shows the per cent scores in CON, dys+, and dys− with a focus on the three domains, which also illustrates differences in task demands within the domains—in Domain 1, T1 > T2; in Domain 2, T4 > T3; in Domain 3, T6 > T7 > T5.

**Figure 3:**
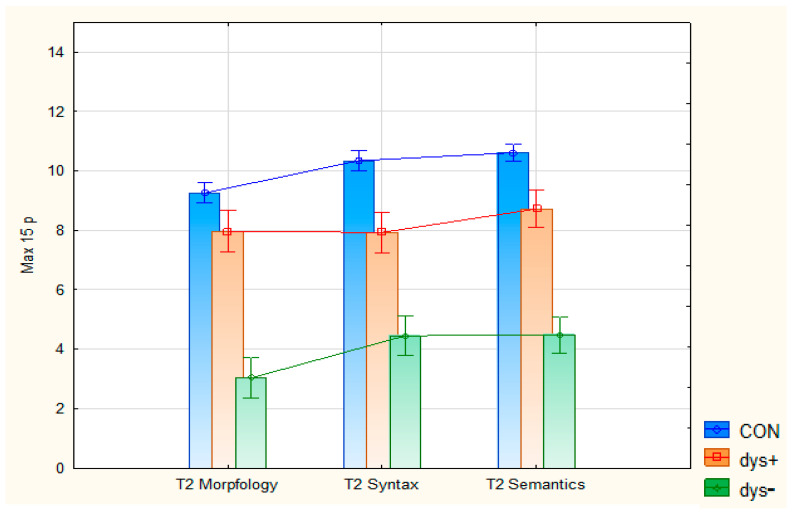
T2 model sentences; additional scoring of morphology, syntax, and semantics.

**Figure 4:**
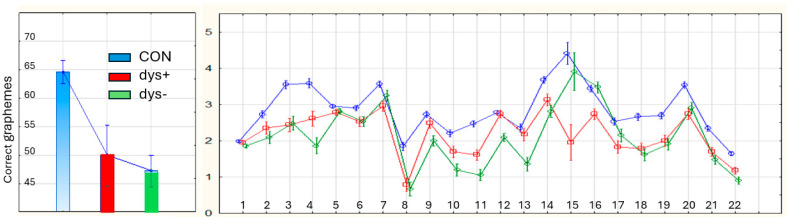
T5 word spelling; additional scoring. Left panel: number of graphemes (means) by group; right panel: graphemes (means) by words and groups. (1) **boy** [2: b-oy], (2) **girl** [3: g-i-rl], (3) **school** [4: s-ch-oo-l], (4) **child** [4: ch-i-l-d], (5) **cat** [3:c-a-t]. (6) **name** [3: n-a-me], (7)**very** [4: v-e-r-y], (8) **should** [3: sh-ou-ld], (9) **nose** [3: n-o-se], (10) **mouth** [3: [m-ou-th], (11) **much** [3: m-u-ch], (12) **when** [wh-e-n]. (13) **could** [3: c-ou-ld], (14) **just** [4: j-u-s-t], (15) **beautiful** [8: b-e-au-t-i-f-u-l], (16) **many** [4: m-a-n-y], (17) **then** [3: th-e-n], (18) **what** [3: wh-a-t], (19) **house** [3: h-ou-se], (20) **little** [4: l-i-tt-l], (21) **than** [th-a-n], (22) **high** [2: h-igh].

Part 2. Additional analyses are shown in Table 4 and Table 5 and in Figure 3 and Figure 4.

**T2 Morphology, Syntax, and Semantics:** (Table 4) analyses of variance (GLM repeated-measures ANOVA).

*CON *vs. *DYS*. showed an effect of group, F_1,184_ = 53.985, *p* < 0.0001, and of repeated measures, F_2,370_ = 21.554, *p* < 0.0001. A follow-up test showed that the effect of group was due to CON (10.073) > DYS (6.028), and that of repeated-measures morphology (8.027) < syntax (8.995) and semantics (9.305), with no difference between syntax and semantics. As shown in Table 4, this pattern was seen separately in both CON and DYS.

**Table 4:** T2 model sentences. Morphology, syntax, and semantics, and T5 word graphemes.

GROUP		CON		DYS		Dys+		Dys−		*t*-Test	One-Way Anova		
Additional Scoring	Max p	Mean	Sd.	Mean	Sd.	Mean	Sd.	Mean	Sd.	CON vs. DYS	CON vs. Dys+	CON vs. Dys−	Dys+ vs. Dys−
**T2**													
**Morphology**	15	9.26	3.99	5.42	4.11	7.97	4.32	3.03	1.91	0.000	0.10	0.000	0.000
**Syntax**	15	10.35	3.68	6.13	3.99	7.93	4.23	4.45	2.93	0.000	0.002	0.000	0.001
**Semantics**	15	10.61	3.38	6.53	3.93	8.72	4.07	4.48	2.45	0.000	0.01	0.000	0.000
**Morph < Syn, Sem**		****		****		ns		*					
**T5**													
**Graphemes**	75	64.60	8.11	48.69	9.75	50.00	12.26	47.26	5.91	0.000	0.000	0.000	0.31

Notes. Repeated-analyses (vertical) measures: morphology (Morph), syntax (Syn), and semantics (Sem). *: *p* < 0.05; ****: *p* < 0.0001; ns: not significant.

*CON *vs. *dys+* vs. *dys−* showed an effect of group: F_2,184_ = 42.535, *p* < 0.0001 and of repeated measures: F_2.368_ = 14.593 *p* < 0.0001. A follow-up test showed that the effect of group was due to CON (10.073) and dys+ (8.207) > dys− (3.989), with no difference between CON and dys+. Repeated measures showed an effect of morphology (8.027) < syntax (8.995) and semantics (9.305), with no difference between syntax and semantics. This pattern was seen in CON and dys−, with no difference between the tests in dys+ (see Table 5).

**Table 5:** Cohen’s d. T2 model sentences and T5 morphology Cohen’s d.

	CON vs. DYS	CON vs. Dys+	CON vs. Dys−	Dys+ vs. Dys−
**T2**				
**Morphology**	0.95	0.38	1.99	1.48
**Syntax**	1.10	0.61	1.77	0.96
**Semantics**	1.11	0.51	2.08	1.26
**T5**				
**Graphemes**	1.77	1.40	2.44	0.28

A similar pattern to the analyses in Table 4 was seen in the effect-size calculation shown in Table 5. In CON vs. DYS, the effect sizes were large overall; in CON vs. dys+, they were low to medium in T2; in CON vs. dys−, the effect sizes were large overall; for dys+ vs. dys−, the effect sizes were large in T2. These results are illustrated in Figure 3.

**T5: word dictation/graphemes.** Each word was scored by correct phoneme/grapheme correspondence. As shown in Table 4, the scores of CON were significantly higher than DYS, dys+, and dys−, with no difference between dys+ and dys−. A similar pattern to the analyses in Table 4 was seen in the effect size calculation in Table 5. All effect sizes were large except for dys+ vs. dys−, which was small.

Figure 4 shows the sum scores for each of the three groups (CON, dys+, and dys−) in the left panel, and the mean scores for the number of graphemes in the right panel.

**Summary, Part 2.** In general, the T2 scores were below the ceiling (15 p) in all group analyses, indicating that this is a demanding part of EFL claiming linguistic awareness. Of the three analyses, morphology proved to be demanding within all groups, but most dominant within dys−.

For T5 graphemes, very few of the 22 words were correctly spelled in the two DYS subgroups. In contrast to all other tasks, the difference between the two subgroups dys+ and dys− on spelling was not significant, and with a small effect size.

## 4. Discussion

In this study, we wanted to explore EFL skills in Norwegian 6th graders with dyslexia compared to a control group. By using the “The English 2 Dyslexia Test” [80], basic verbal, pragmatic, and literacy skills were assessed in two parts. Part 1 assesses verbal and literacy skills, while Part 2 assesses basic linguistic and orthographic skills. As mentioned in the Introduction, these elements can be analysed separately, although they mutually influence each other. This was manifested by the significant correlations between the scores of the seven tests.

In Part 1, the first focus was on group differences. As expected, the dyslexia group scored significantly lower than the control group on all tasks. However, the variations within the dyslexia group were larger than expected. The splitting on comprehension scores created a subgroup (dys+) that performed nearly as well as the control group on several of the tests, and a subgroup (dys−) that scored significantly below both the control group and the dys+ group on most tests, except for spelling, where minor differences to dys+ were seen. This parallels our earlier findings in which subgrouping was based on L1 comprehension [47].

The second focus in Part 1 was on categorization of the seven tests into three domains, which revealed differences within each domain regarding task demands. The four high-score tasks (T1, T4, T6, and T7) create major demands for top-down processing, i.e., content and use, while three low-score tasks (T2, T3, and T5) create major demands on bottom-up processing, i.e., form [39,48]. In other words, the tasks put different demands at the cognitive level, as described in the Introduction [12,13,82] and in line with the cognitive benchmarks in bilingual dyslexia: phonological processing, auditory system, visual system, processing speed, and semantic lexicon [29,83].

A core finding of Part 1 was that the subgrouping demonstrates substantial variations within dyslexia. Dys+ managed four top-down tasks nearly in line with the control group, and with relatively high scores on two of the literacy tests, reading (T6) and translation (T7). This indicates good functions within the phonological system in this subgroup. That dys− scored low on all seven tasks indicates major problems within the working memory system, especially auditory processing, but it might be combined with visual processing problems as well.

In Part 2, this discrepancy was further assessed by detailed analyses on bottom-up processing, i.e., on form. The T2 model sentences are demanding on several elements within verbal working memory: first, the reader has to remember the presented picture and the adjacent verbal sentence, then keep the verbal syntax pattern in mind to comply and adjust semantics and morphology to the following presented picture. Most mistakes were seen in the morphology category, but with minor differences between the control group and dys+, and with major differences to the dys− subgroup.

The Domain 3 Literacy tests focus on 22 high-frequency words that the subjects had heard, seen, read, and written many times during their six years of EFL training. Interestingly, the T6 reading and T7 translation scores indicated familiarity and comprehension to a certain degree for all participants, but this was not reflected in T5 spelling. The very low spelling scores in both dyslexia subgroups show that the visual form of the words has not been stored in or recalled properly from long-term memory. Instead, an effort may have been put into a letter/sound combination in line with the subjects’ L1 learning, without being able to recall the correct grapheme. This strengthens our assumption that the spelling problems in dys+ are mainly related to impaired visual processing, while all three literacy scores in dys− are related mainly to poor auditory processing and possibly to poor visual processing. Thus, our second core finding in this study is in line with studies showing that dyslexia is caused by impaired timing signalling of visual and auditory cues essential for reading [21,84].

In conclusion, these divergent visual and auditory processing profiles align with neurocognitive models of dyslexia that implicate disrupted temporal coordination in auditory and visual processing streams. No comprehension problems but impaired spelling in dys+ indicate intact auditory–phonological language streams. Most likely, the scores of the dys− subgroup across verbal and literacy tasks suggest atypical functioning in the left-hemisphere dorsal and ventral language streams.

### 4.1. Limitations of the Study and Further Research

This study is a cross-sectional study and gives no information on how EFL skills in students with dyslexia develop. Also, the sample sizes in the dyslexia subgroups are small, and therefore, the results must be interpreted with care. Longitudinal studies and/or cross-sectional studies at the different literacy stages provide valuable information about EFL for both typical students and students with dyslexia. Important for the interpretation of the subgroup differences is access to professional assessment reports and diagnostic profiles of the dyslexia participants, which was not an option in the present study.

Most important in further studies is to link behavioural patterns to neural systems. Future EFL studies should integrate neuroimaging (e.g., fMRI, EEG, and MEG) to directly test whether the impaired behavioural profiles found in the present study are reflected in impaired neurolinguistic patterns related to left-hemisphere language networks, to auditory and visual timing systems, and to working memory components such as the phonological loop and the visuo-spatial sketchpad. Moreover, it is important to assess how these patterns may relate to neural correlates of bilingualism and dyslexia, as described in cross-linguistic studies [20,85].

The consequences of low EFL comprehension lead to the question of comorbidities. Many individuals with dyslexia have a history of Developmental Language Disorder (DLD), affecting early language development in both comprehension and production [86,87,88]. The EFL language problems seen in dys− especially align with language problems seen in DLD in L1. None of the participants in this study had any reported comorbidities, and low comprehension and pragmatic scores were related to neurolinguistic problems within working memory, which is a core benchmark in DLD. As mentioned in the Introduction, studies show that children’s difficulties in their L1 mirror their L2 learning, especially in literacy, vocabulary, and working memory [46]. DLD is usually diagnosed in the pre-literacy stage, and studies have shown weak L2 skills in DLD [89,90]. However, studies on EFL in dyslexia combined with DLD seem scarce, calling for more research within this area.

At the environmental level, two other topics need more research: first, the cracking of the literacy code in L1 and learning EFL at the same time; second, the competence of EFL teachers in primary school. In Denmark, English was introduced in 1st grade as late as 2014. This sparked a discussion of whether an early start leads to increased EFL learning, about the organization of teaching, about teachers’ competence, and about didactics [91]. Cadierno et al. [68] compared EFL competence among a group of Danish pupils who started EFL learning in 1st grade with a class that started in 3rd grade. They concluded that the students who started EFL in 3rd grade did better than those who started in 1st grade. At a biological level, following up on studies of brain lateralization in mono- and bilinguals [24,25] may shed more insight into the effects of simultaneous L1 literacy learning and EFL learning in dyslexia.

Also, the effect of gaming on EFL proficiency has been shown [73,74,75]. A systematic literature review on gaming and EFL among students aged 11–18 years concluded with increased motivation and learning [71]. Time spent and gender differences in relation to this activity provide knowledge about how gaming affects the EFL skills in students with dyslexia. Such behavioural studies should integrate neuroimaging not only to investigate the effects of gaming, but also how programs can be designed and used in accordance with individual dyslexic profiles.

However, some warning signs regarding digitalization should not be overlooked. Current research indicates that even after controlling for social background, high use of screen time in early childhood is longitudinally associated with poorer developmental outcomes in terms of language, learning, and social skills [92]. Also, there is a challenge in that Artificial Intelligence (AI) can take over any EFL communication. This can be tempting for students with dyslexia, especially, and therefore, more knowledge is needed about digital programs and AI, both as facilitators and inhibitors of EFL in dyslexia.

In sum, brain studies involving comorbidities, age, and digitalization are future challenges in EFL dyslexia research.

### 4.2. Relevance to Practice

It is noted that the field of dyslexia and L2 assessments is growing, but more research is needed [93]. A qualified L1 dyslexia assessment will describe a cognitive profile to direct further intervention methods, which, according to our findings, should include verbal as well as literacy training. Students with dyslexia can and should learn foreign languages on an equal footing with their peers. However, in line with L1 teaching, EFL teaching must be adjusted, preferably in collaboration with professionals with special expertise in dyslexia. In any case, the strengths and weaknesses of the subject must be mapped, and the teaching should be based on continuous evaluation. For instance, if a professional dyslexia assessment reveals problems within auditory working memory, often described as phonological processing problems, intervention should focus on remediation and compensation within this domain. On the other hand, if the problems are within visual working memory, the intervention should focus on this domain and not on phonological processing, which is often the case when dyslexia is defined solely as a phonological impairment.

### 4.3. Concluding Remarks

The core finding of this study was that two different dyslexia profiles emerged from the verbal and literacy EFL testing. One profile showed minor problems with verbal skills, but major problems with spelling, indicating visual processing problems. The other profile showed major problems with both verbal and literacy skills, indicating impairment within auditory processing and possibly also within visual processing. How individuals with dyslexia learn a new language can provide insight into the understanding of what dyslexia is. In this project, the meeting point was between two relatively compatible language typologies, but with substantial differences in orthography. Our knowledge of EFL in dyslexia calls for more research. The results from the present study point specifically to more research on how the behavioural patterns in the two dyslexia subgroups relate to neural correlates in the meeting between EFL and different L1 language typologies and orthographies.

## Figures and Tables

**Table 1:** Participants by groups and gender.

	Total	CON		DYS		Dys+		Dys−	
	N	N	M/F	N	M/F	N	M/F	N	M/F
**Dom 1 and 3**	187	127	63/64	60	26/34	29	15/14	31	11/20
Chi^2^			ns		ns		ns		ns
Dom 2	147	99	47/52	48	21/27	27	14/13	21	7/14
Chi^2^			ns		ns		ns		ns
Points			251/230		194/171		214/198		155/147
LSD			ns		ns		ns		ns

Note: Points = sum of all test scores (max sum = 442). Chi^2^ and LSD follow-up test showed no statistical differences in gender, groups, or total score; ns: not significant.

**Table 2:** Descriptive data by groups and group analyses.

GROUP		CON		DYS		Dys+		Dys−		*t*-Test	One-Way Anova		
Main Variables	Max p	Mean	Sd.	Mean	Sd.	Mean	Sd.	Mean	Sd.	CON vs. DYS	CON vs. Dys+	CON vs. Dys−	Dys+ vs. Dys−
**D1 Sentences**													
T1 Lang Comp.	15	12.72	1.94	11.03	2.48	!3.14	1.94	9.06	1.59	0.000	0.26	0.000	0.000
T2 Model sent.	15	7.05	3.79	3.20	3.41	5.21	3.74	1.32	1.54	0.000	0.01	0.000	0.000
*T1* > *T2*		****		****		****		****					
**D2 Pragmatics**													
T3 Convers.	173	69.69	19.46	57.17	19.89	62.11	18.86	50.81	19.79	0.000	0.07	0.000	0.05
T4 Pict. story	173	95.06	33.57	69.72	37.48	82.30	37.44	53.55	31.48	0.000	0.09	0.000	0.005
*T3* < *T4*		****		*		**		ns					
**D3 Literacy**													
T5 Word dict.	22	14.38	5.06	7.03	4.61	8.14	5.82	6.00	2.80	0.000	0.000	0.000	0.09
T6 Reading	22	20.32	1.94	18.00	2.91	19.07	2.95	17.00	2.52	0.000	0.007	0.000	0.000
T7 Translation	22	19.18	2.57	14.58	5.16	16.41	5.35	12.87	4.41	0.000	0.000	0.000	0.000
*T5* < *T6*, *T7*		****		****		****		****					
*T7* < *T6*		*		****		**		****					

Notes: D = domain. Repeated-measures analyses within each domain (vertical) *: *p* < 0.05; ** *p* < 0.01; ****: *p* < 0.0001; ns: not significant.

**Table 3:** Cohen’s d from descriptive data by groups.

Domains		CON vs. DYS	CON vs. Dys+	CON vs. Dys−	Dys+ vs. Dys−
D1	T1	0.81	0.21	1.97	2.31
	T2	1.05	0.49	1.65	1.38
D2	T3	0.64	0.39	0.97	0.59
	T4	0.73	0.37	1.24	0.82
D3	T5	1.49	1.20	1.78	0.47
	T6	1.01	0.58	1.61	0.76
	T7	1.28	0.85	2.10	0.72

## Data Availability

The data presented in this study are not available at this point due to ethical standards.
